# Respiratory Variations in Pulse Pressure Reflect Central Hypovolemia during Noninvasive Positive Pressure Ventilation

**DOI:** 10.1155/2014/712728

**Published:** 2014-02-19

**Authors:** Ingrid Elise Hoff, Lars Øivind Høiseth, Jonny Hisdal, Jo Røislien, Svein Aslak Landsverk, Knut Arvid Kirkebøen

**Affiliations:** ^1^Norwegian Air Ambulance Foundation, Holterveien 24, 1441 Drøbak, Norway; ^2^Department of Anaesthesiology, Oslo University Hospital, P.O. Box 4956, Nydalen, 0424 Oslo, Norway; ^3^Faculty of Medicine, University of Oslo, P.O. Box 1072 Blindern, 0316 Oslo, Norway; ^4^Department of Vascular Medicine, Oslo University Hospital, P.O. Box 4956, Nydalen, 0424 Oslo, Norway; ^5^Department of Biostatistics, Institute of Basic Medical Sciences, University of Oslo, P.O. Box 1072 Blindern, 0316 Oslo, Norway

## Abstract

*Background*. Correct volume management is essential in patients with respiratory failure. We investigated the ability of respiratory variations in noninvasive pulse pressure (ΔPP), photoplethysmographic waveform amplitude (ΔPOP), and pleth variability index (PVI) to reflect hypovolemia during noninvasive positive pressure ventilation by inducing hypovolemia with progressive lower body negative pressure (LBNP). *Methods*. Fourteen volunteers underwent LBNP of 0, −20, −40, −60, and −80 mmHg for 4.5 min at each level or until presyncope. The procedure was repeated with noninvasive positive pressure ventilation. We measured stroke volume (suprasternal Doppler), ΔPP (Finapres), ΔPOP, and PVI and assessed their association with LBNP-level using linear mixed model regression analyses. *Results*. Stroke volume decreased with each pressure level (−11.2 mL, 95% CI −11.8, −9.6, *P* < 0.001), with an additional effect of noninvasive positive pressure ventilation (−3.0 mL, 95% CI −8.5, −1.3, *P* = 0.009). ΔPP increased for each LBNP-level (1.2%, 95% CI 0.5, 1.8, *P* < 0.001) and almost doubled during noninvasive positive pressure ventilation (additional increase 1.0%, 95% CI 0.1, 1.9, *P* = 0.003). Neither ΔPOP nor PVI was significantly associated with LBNP-level. *Conclusions*. During noninvasive positive pressure ventilation, preload changes were reflected by ΔPP but not by ΔPOP or PVI. This implies that ΔPP may be used to assess volume status during noninvasive positive pressure ventilation.

## 1. Introduction

Pulse pressure variations (ΔPP) reflect volume status during mechanical ventilation [1]. Respiratory variations in the photoplethysmographic waveform amplitude (ΔPOP) and the pleth variability index (PVI) are proposed as noninvasive alternatives [2, 3]. In spontaneously breathing subjects, only passive leg raise and the end-expiratory occlusion test have been shown to consistently reflect preload dependency [4, 5]. However, the literature is divided on two major issues concerning the usefulness of dynamic variables: whether they are applicable during spontaneous breathing [6, 7] and whether the photoplethysmographic waveform derived variables are useful alternatives to pulse pressure variation [8, 9]. Due to this uncertainty, the applicability of dynamic variables is currently limited to patients on controlled mechanical ventilation. However, with increasing data on negative consequences of intubation and mechanical ventilation, noninvasive positive pressure ventilation (NPPV) is frequently used in emergency departments and intensive care units. Patients treated with NPPV are often on the verge of respiratory failure, and correct fluid management is essential.

The aim of the present study was therefore to explore the ability of dynamic variables to reflect graded hypovolemia during NPPV. Lower body negative pressure (LBNP) is a well-established model for central hypovolemia and preload reduction [10, 11]. This noninvasive model in healthy volunteers enables investigation of arterial pressure and photoplethysmographic waveform derived variables without pain or medication, factors which could influence the results. We hypothesized that the reduction in stroke volume (SV) induced by increasing levels of LBNP would be aggravated with the application of NPPV and that the reduction in SV would be reflected in the dynamic variables ΔPP, ΔPOP, and PVI.

## 2. Materials and Methods

After approval by the regional ethics committee (REK Sør-Øst, ref.no 2009/2180, December 2009), written informed consent was obtained from 14 healthy volunteers (7 male, 7 female, aged 28 ± 7 years, height 177 ± 10 cm, and weight 71 ± 13 kg (mean ± SD)). The subjects were instructed to refrain from alcohol or caffeinated drinks 24 hours prior to participation. Pregnant women and subjects using cardiovascular medication were not included.

### 2.1. Experimental Protocol

**Figure 1:**
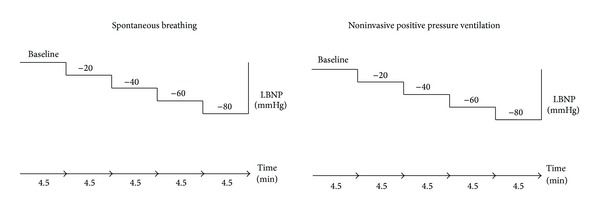
Schematic illustration of the experimental protocol. Each level was kept for 4.5 min. LBNP: lower body negative pressure.

Subjects were in the supine position during experiments, which were performed in room temperature. LBNP was applied by a custom made LBNP-chamber previously described [12] and induced by stepwise suction of air out of the chamber. After baseline measurements, subjects underwent consecutive LBNP-pressures of −20, −40, −60, and −80 mmHg. Each level was kept for 4.5 min. After minimum 15 min rest, the procedure was repeated with NPPV. NPPV was applied via a face mask with intermittent positive pressure (IPPV-mode), tidal volume 10 mL/kg ideal weight, positive end-expiratory pressure (PEEP) = 0 cm H_2_O, fraction of inspired oxygen 0.21, and respiratory frequency of 10–12/min (Evita 4, Dräger Medizintechnik GmbH, Lübeck, Germany). Spontaneous breathing and mask leakage were minimized by thorough mask adjustment and by ensuring compliance with the ventilation mode before data recordings. The protocol was discontinued if one of the following events occurred: systolic blood pressure <70 mmHg, a sudden decrease in systolic blood pressure ≥15 mmHg, a decrease in heart rate (HR) ≥15 beats/min, dizziness, sweating, or nausea. The experimental protocol is illustrated in Figure 1.

### 2.2. Data Acquisition and Analysis

Data were recorded over the total interval of each LBNP-level, that is, 4.5 min. Data from all completed LBNP-levels are included in the analysis. Calculations were made from data sampled and averaged over 10 consecutive respiratory cycles without arrhythmia. Respiratory movements were recorded with a custom-made air flowmeter. During NPPV tidal volume, respiratory rate, airway pressures, leakage, and spontaneous breathing activity were continuously measured and recorded every 10 s using commercial software (VentView 2.0, Dräger Medical Ag & Co, Lübeck, Germany). In order to investigate the effects of NPPV the data was manually filtered after recording and 10 consecutive respiratory cycles (1 min) without excessive spontaneous breathing (>5% of the corresponding minute volume per respiratory cycle) were identified. Hemodynamic measurements from the corresponding minute were then analyzed. ΔPP and ΔPOP were calculated in a custom made program (Labview 8.2; National Instruments, TX, USA), according to Michard [13]. One respiratory cycle was manually delimitated and the program displayed corresponding blood pressure and photoplethysmographic waveform curves. The photoplethysmographic waveform, PVI, and PI were obtained from a finger clip (Masimo Radical 7, version 7.3.1.1, Masimo Corp., Irvine, CA, USA) on the third finger of the right hand, which was covered to prevent temperature loss and disturbance of the signal from ambient light. PVI and PI were calculated according to the manufacturer's algorithms (http://www.masimo.com/pdf/whitepaper/LAB4583A.pdf; 13.09.2013). Averaging period for PI was set to 2 s in the pulse oximeter. PVI and PI were downloaded using the TrendCom software (Masimo) and averaged over 1 min. Continuous arterial pressure was obtained noninvasively at heart level from the left third finger (Finometer, FMS Finapres Medical Systems BV, Amsterdam, The Netherlands). SV was obtained continuously with suprasternal Doppler (SD-100, GE Vingmed Ultrasound, Horten, Norway) by an experienced operator. An angle of 20° and a diameter of the aortic valve of 20 mm were assumed in the calculation of SV from the velocity-time integrals. Heart rate (HR) was obtained from a standard 3-lead electrocardiogram (ECG). ECG, arterial pressure, and photoplethysmographic waveforms were sampled at 400 Hz.

### 2.3. Statistics

The primary endpoint was the change in ΔPP, ΔPOP, PVI, and PI following the transition from spontaneous breathing to noninvasive positive pressure ventilation. Data are given as mean ± SD unless otherwise stated. The associations between the independent variables LBNP and NPPV and the dependent variables HR, mean arterial pressure (MAP), SV, ΔPP, ΔPOP, PVI, and PI were analyzed by linear mixed model regression analyses. Linear mixed model is a generalization of traditional linear regression, which adjusts for the correlation between repeated measurements within each subject and finds the best linear fit to the data across all individuals. The model maximizes power by utilizing all data despite missing observations following the premature termination of the LBNP-protocol in some subjects. The effect estimates describe the mean effect of LBNP on the hemodynamic variables when going from one LBNP-level to the next. The difference in hemodynamic variables with and without NPPV was assessed by adding the interaction term LBNP∗NPPV in the regression model. Introduction of an interaction term is necessary where the effect of one variable (LBNP) is affected by the presence or value of another variable (NPPV). Results are given as coefficients (beta-values) with 95% confidence intervals (CI). *P* values below 0.05 were considered statistically significant. As no similar studies had previously been published, a conventional power analysis was not performed. Due to the experimental nature of the study, the number of study subjects was limited to 14. Statistical calculations were performed in SPSS 19.0 (SPSS Inc., Chicago, IL, USA) and R 2.12 (R Foundation for Statistical Computing, 2011).

## 3. Results

**Figure 2:**
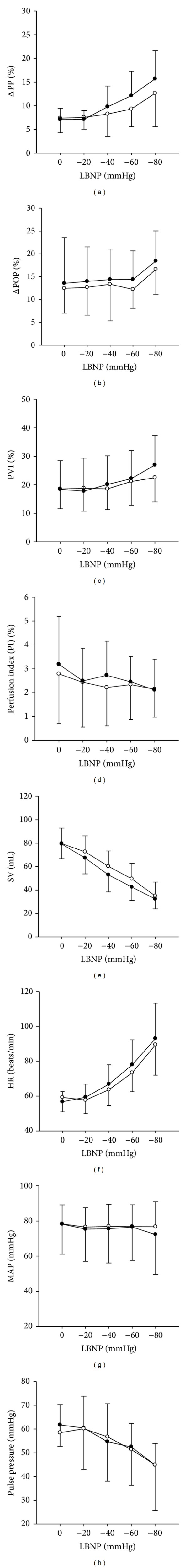
Line charts of mean values at each LBNP-level for ΔPP, ΔPOP, PVI, PI, SV, HR, MAP, and PP. Open circle: measurements during spontaneous breathing. Full circle: measurements during NPPV. 1 SD illustrated with one-sided error bars for clarity. ΔPP: respiratory variations in pulse pressure, ΔPOP: respiratory variations in the photoplethysmographic waveform amplitude, PVI: pleth variability index, PI: perfusion index, SV: stroke volume, HR: heart rate, MAP: mean arterial pressure, PP: pulse pressure, NPPV: noninvasive positive pressure ventilation, LBNP: lower body negative pressure.

**Figure 3:**
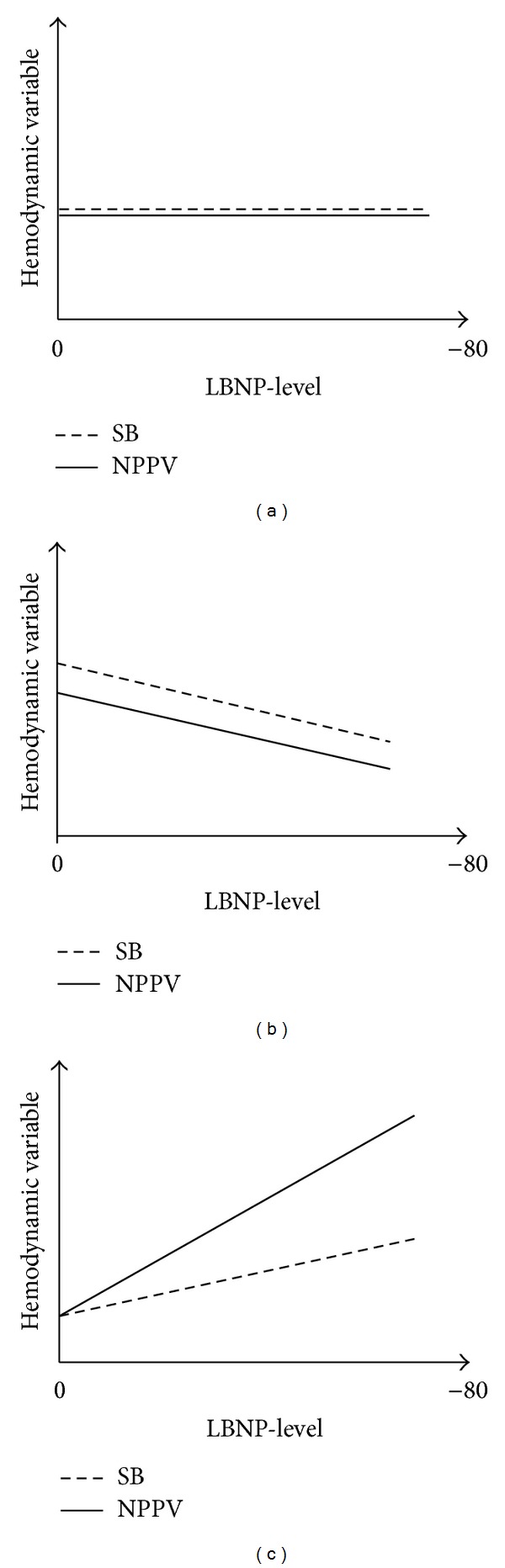
Illustration of different effects of LBNP and NPPV on hemodynamic variables. (a) No effect of LBNP or NPPV alone or in combination (for instance MAP), (b) independent effects of both LBNP and NPPV (stroke volume), and (c) independent effect of LBNP which increases in combination with NPPV (interaction; ΔPP). No effect of NPPV alone. LBNP: lower body negative pressure, NPPV: noninvasive positive pressure ventilation, SB: spontaneous breathing, MAP: mean arterial pressure, ΔPP: pulse pressure variation.

The number of subjects completing each level is shown in Table 1. Two subjects failed to comply with NPPV despite successful testing but completed the LBNP-series during spontaneous breathing. Data from these two subjects are included in the analysis of the effect of LBNP on hemodynamic variables. Hemodynamic data are summarized in Table 1 and mean values shown in Figure 2. Results from the linear mixed model analyses are given in Table 2. Different effects of LBNP and NPPV on hemodynamic variables are illustrated in Figure 3.

SV decreased significantly with progressive levels of LBNP, with a mean reduction of 11.2 mL (95% CI −11.8, −9.6, *P* < 0.001) between each LBNP-level. After application of NPPV, SV was significantly lower at all LBNP-levels (−3.0 mL, 95% CI −8.5, −1.3, *P* = 0.009) compared to spontaneous breathing (Figure 3(b)). ΔPP was significantly affected both by LBNP-level alone and by the interaction between LBNP and NPPV (Figure 3(c)). Whereas ΔPP increased by 1.2% (95% CI 0.5, 1.8, *P* < 0.001) between each LBNP-level during spontaneous breathing, the application of NPPV led to an additional increase of 1.0% (95% CI 0.1, 1.9, *P* = 0.033) during LBNP, almost a doubling. NPPV alone did not affect ΔPP significantly, meaning that NPPV only led to an increase in ΔPP during hypovolemia induced by LBNP. Neither LBNP nor NPPV altered ΔPOP or PVI significantly. PI decreased significantly with progressive LBNP-levels, but was not affected by NPPV. Heart rate was significantly affected by LBNP alone. MAP did not change significantly during the LBNP-protocol or following the transition from spontaneous breathing to NPPV (Figure 3(a)). Pulse pressure decreased significantly between each LBNP-level (−3.5 mmHg, 95% CI −4.5, −2.0, *P* < 0.001) but was unaffected by NPPV.

## 4. Discussion

The main finding in this experimental study is that ΔPP consistently reflected progressive central hypovolemia during NPPV. The application of NPPV accentuated the reduction in SV induced by LBNP. This accentuation was reflected by ΔPP but not by ΔPOP or PVI. There were no associations between LBNP-levels and ΔPOP or PVI. Neither ΔPOP nor PVI changed significantly with the transition from spontaneous ventilation to NPPV.

### 4.1. SV and Pulse Pressure

The effects of LBNP on neurohumoral and sympathetic neural activity have been thoroughly described [10]. Increased vasomotor tone during early hypovolemia preserves systemic blood pressure and may in combination with increased heart rate mask reduced SV. We observed this compensatory response in the present study. Pulse pressure decreased with progressive LBNP-levels, but the decrease in pulse pressure occurred later than the decrease in SV. Similar results were reported in another study on spontaneously breathing volunteers undergoing progressive LBNP [14]. NPPV leads to an accentuated reduction in SV at all levels of LBNP except baseline. This is in accordance with the known impact of increased intrathoracic pressure on venous return during central hypovolemia [15]. The opposite effect was demonstrated by Ryan et al. [16] who found that breathing through an inspiratory threshold device preserved SV by reducing intrathoracic pressure, which increased LBNP-tolerance in healthy volunteers.

### 4.2. ΔPP, ΔPOP, PVI, and PI

Hemodynamic effects of controlled mechanical ventilation [10] and the ability of ΔPP to reflect hypovolemia during mechanical ventilation have previously been demonstrated [17]. There are also studies indicating that dynamic variables like stroke volume variation and vena collapsibility index may be useful during spontaneous respiration [6]. Heart-lung interactions differ substantially between spontaneous breathing and mechanical ventilation, and insufficient changes in intrathoracic pressure due to low, irregular tidal volumes and irregular respiratory rates impede the use of respiratory induced variables to evaluate preload or preload dependency. Whereas mechanical inspiration reduces right ventricular filling and increases right ventricular afterload, spontaneous inspiration increases both right ventricular filling and right ventricular afterload. Deep inspiration could possibly induce sufficient changes in intrathoracic pressure to be reflected in SV and pulse pressure. Préau et al. [18] found that ΔPP predicted fluid responsiveness in spontaneously breathing patients undergoing a deep inspiratory maneuver but with lower sensitivity than reported in studies on mechanically ventilated patients. Similarly, Soubrier et al. [19] tested volume responsiveness in spontaneously breathing patients and found that ΔPP and variations in systolic blood pressure at baseline were significantly higher in responders than in nonresponders, but sensitivity was low. In a study on spontaneously breathing pigs ΔPP was significantly higher during hypovolemia, but ΔPP only predicted fluid responsiveness when an expiratory resistor was added [20]. Whereas none of these studies show that ΔPP predicts fluid responsiveness with sufficient sensitivity, they indicate that pulse pressure variations may reflect hypovolemia during spontaneous breathing. This is in line with our findings, but in addition we show how ΔPP increases following the transition from spontaneous breathing to NPPV. The substantial increase in ΔPP during NPPV indicates that NPPV alters intrathoracic pressure similarly to invasive mechanical ventilation, provided compliance with the ventilator and minimal spontaneous breathing. Heenen et al. investigated dynamic variables in patients with spontaneous breathing movements and found that ΔPP varied substantially at baseline with no significant differences between fluid responders and nonresponders, and areas under the receiver operating characteristics curves were low [7]. No standardization of respiratory rate, tidal volumes, or airway pressure was attempted in this study, which may in part explain their results. By manually filtering the data after recordings we were able to investigate the effects of NPPV alone, undisturbed by excessive spontaneous breathing.

There are conflicting reports on ΔPOP as an alternative to ΔPP to reflect hypovolemia and predict fluid responsiveness [8, 21]. Differences in vasomotor tone, measurement sites, and measurement methodology contribute to discrepant results. Promising studies on variables derived from the photoplethysmographic waveform have mainly been performed in stable patients during anesthesia, over short periods of time, and in the absence of sympathetic triggers such as advanced hypovolemia, surgery, pain, and stress [8, 22]. In mechanically ventilated patients undergoing stepwise blood withdrawal, Pizov et al. [17] found that arterial waveform variables detected mild hypovolemia earlier and more consistently than photoplethysmographic variables. Interestingly, correlations between ΔPOP and ΔPP improved with increasing hypovolemia of up to 20% of estimated blood volume, with ΔPOP showing the largest variability. These were all stable patients with no signs of circulatory failure. As shown in Table 1 and Figure 2, we found that ΔPOP increased with progressive LBNP, but due to large confidence intervals this increase was not statistically significant. Other studies have demonstrated wide limits of agreement between ΔPP and ΔPOP during surgery and intensive care [9, 21, 23, 24]. The physiology behind the photoplethysmographic signal is very complex, influenced by cardiac and autonomic as well as respiratory factors [25]. Due to rich innervation and large vascular plexuses the finger is very sensitive to vasomotion [26]. Using spectral analysis on both spontaneously breathing and mechanically ventilated patients, Shelley et al. [27] found a correlation between estimated blood loss and ventilatory effects in the ear signal but not in the finger signal. We believe that increased sympathetic activation leading to vasoconstriction is the main reason why ΔPOP to a lesser extent than ΔPP reflected hypovolemia in our study and largely explains the discrepancy between this study and others performed on stable patients during normovolemia.

In addition to different measurement sites, the use of different features of the photoplethysmographic signal may explain conflicting reports. In a LBNP-model, McGrath et al. [28] studied the correlation between changes in SV and changes in pulse width, amplitude, and area of an unfiltered signal and found that it differed for all measurement sites. The lowest correlation was between changes in SV and pulse amplitude in the finger, the feature most commonly used when calculating respiratory changes in the photoplethysmographic waveform. Commercial finger pulse oximeters are frequently used in clinical practice and should be investigated. However, probes from different manufacturers could also contribute to different results as sensitivity and signal processing differ [29]. We obtained both pulse pressure and photoplethysmographic waveforms from noninvasive finger probes. The two measurement methods are based on different physiological principles (volume measurements in relatively large finger arteries versus absorption of infrared light from the tissues, primarily reflecting microcirculatory changes). It has previously been described how the complexity of the photoplethysmographic signal renders it more susceptible to “noise” which may be physiological or technological artifacts influencing the signal [29]. Our findings are in line with this explanation.

There was no significant relationship between PVI and LBNP-level in our study. Like other features of the photoplethysmographic waveform PVI depends on stable perfusion and vasomotion, as demonstrated in a recent study where changes in vasomotor tone were induced by norepinephrine [30]. Another study showed that PVI failed to predict fluid responsiveness in mechanically ventilated patients when PI was low (<4), whereas pulse pressure variations reliably did [31]. The PI value depends on the fraction of infrared light returning from the measurement site and represents the ratio of pulsatile (pulsating blood) to nonpulsatile (nonpulsating blood, bone, and soft tissue) signal. PI is therefore affected by changes in vasomotor tone as this affects the ratio between pulsatile and nonpulsatile blood [32]. Increased vasomotor tone and low SV may explain the reduction in PI in the present study, and PI has been suggested as an early marker of the physiologic responses that occur during hypovolemia [33]. We experienced temporary loss of PVI signals in several subjects both during normo- and hypovolemia, although the photoplethysmographic waveform remained well defined. PI was <1 in all cases. The loss of PVI-signal has been described earlier [3].

### 4.3. Methodological Considerations

First, even with measurements on several levels in each subject our sample size is limited. It is however comparable to other studies using the LBNP-model [34, 35] and we were able to demonstrate significant effects of both hypovolemia and NPPV on SV and ΔPP. Second, we wanted to investigate the physiology in a completely noninvasive experimental model in order to avoid disturbing factors such as pain, agitation, and medication. Whereas pulse pressure variations are normally investigated using an invasive line, the Finapres technology has been validated for arterial pressure waveform analysis [21, 36] and blood pressure measurements [37]. Third, while the LBNP-model allows investigation of reversible central hypovolemia, it is not suitable for conventional fluid responsiveness testing using intravenous fluid boluses. However, in this model fluid responsiveness is “tested” by termination of LBNP, which inevitably leads to increased preload and the restoration of hemodynamic variables [10]. Finally, application of NPPV to healthy, nonsedated subjects is challenging. The major issues were to minimize spontaneous breathing and leakage. High tidal volumes, PEEP = 0 and IPPV-mode proved necessary to ensure compliance with NPPV and keep tidal volumes stable. Despite a compensatory increase in frequency, a reduction in tidal volumes to 8 mL/kg increased spontaneous breathing efforts and leakage. Previous studies indicate that airway pressures, ventilation mode, tidal volumes, and lung compliance affect the hemodynamic effects of mechanical ventilation and thus the performance of dynamic variables [38–40]. Spontaneous breathing efforts varied in and between subjects and might have affected central hemodynamics. However, as we filtered the respiratory data obtained during NPPV after recording we identified periods with high, stable tidal volumes that were sufficient to result in measurable preload changes.

### 4.4. Conclusion

The main new finding in this experimental study on central hypovolemia is that ΔPP, but not ΔPOP or PVI, is significantly associated with LBNP-level in healthy volunteers during NPPV. Clinically, this implies that ΔPP may be used to evaluate volume status in patients treated with NPPV. Further, clinical studies are needed to clarify the potential for ΔPP in this setting.

## Figures and Tables

**Table 1:** Dynamic variables and hemodynamic data during spontaneous breathing and noninvasive positive pressure ventilation.

LBNP-level	Subjects completing the LBNP-level (*n*)		ΔPP (%)		ΔPOP (%)		PVI (%)		PI (%)	
	SB	NPPV	SB	NPPV	SB	NPPV	SB	NPPV	SB	NPPV
Baseline	14	12	7.4 ± 3.1	7.1 ± 2.4	12.4 ± 5.4	13.5 ± 10.0	18.5 ± 6.9	18.4 ± 10.0	2.8 ± 2.1	3.2 ± 2.0
20	14	12	7.6 ± 2.5	7.1 ± 1.9	12.7 ± 6.1	14.0 ± 7.5	18.8 ± 8.0	17.8 ± 11.6	2.4 ± 1.9	2.5 ± 1.4
40	13	10	8.3 ± 8.0	9.8 ± 4.4	13.4 ± 8.0	14.4 ± 6.7	18.6 ± 7.2	20.1 ± 10.1	2.2 ± 1.6	2.7 ± 1.4
60	12	10	9.3 ± 3.8	12.1 ± 5.2	12.2 ± 4.1	14.4 ± 6.2	21.1 ± 8.3	22.1 ± 9.9	2.3 ± 1.4	2.5 ± 1.1
80	11	9	12.6 ± 7.1	15.7 ± 6.0	16.6 ± 5.5	18.4 ± 6.6	22.5 ± 8.5	26.9 ± 10.4	2.1 ± 1.2	2.1 ± 1.3

LBNP-level	Subjects completing the LBNP-level (*n*)		SV (mL)		HR (beats/min)		MAP (mm Hg)		PP (mm Hg)	
	SB	NPPV	SB	NPPV	SB	NPPV	SB	NPPV	SB	NPPV

Baseline	14	12	80 ± 13	79 ± 12	59 ± 8	57 ± 6	78 ± 11	78 ± 17	58 ± 12	62 ± 9
20	14	12	73 ± 14	67 ± 14	58 ± 8	59 ± 8	76 ± 11	75 ± 18	60 ± 14	60 ± 17
40	13	10	60 ± 13	53 ± 15	64 ± 9	67 ± 11	77 ± 13	76 ± 20	57 ± 14	55 ± 17
60	12	10	50 ± 13	43 ± 11	73 ± 11	78 ± 14	77 ± 12	77 ± 19	51 ± 11	52 ± 16
80	11	9	35 ± 12	32 ± 8	89 ± 17	93 ± 20	77 ± 14	72 ± 23	45 ± 9	45 ± 19

Data are mean ± SD. LBNP: lower body negative pressure; SB: spontaneous breathing; NPPV: noninvasive positive pressure ventilation; ΔPP: pulse pressure variation; ΔPOP: photoplethysmographic waveform variation; PVI: pleth variability index; PI: perfusion index; HR: heart rate; SV: stroke volume; MAP: mean arterial pressure; PP: pulse pressure.

**Table 2:** Data from the generalized mixed model analyses showing effect estimates of LBNP and NPPV separately and in combination (LBNP ∗ NPPV).

	ΔPP (%)		ΔPOP (%)		PVI (%)		PI (%)	
	Estimate (95% CI)	*P* value	Estimate (95% CI)	*P* value	Estimate (95% CI)	*P* value	Estimate (95% CI)	*P* value
LBNP	1.2 (0.5, 1.8)	<0.001^#^	0.7 (−0.3, 1.3)	0.233	1.0 (−0.5, 1.4)	0.337	−0.1 (−0.4, 0.0)	0.018^#^
NPPV	−0.6 (−2.7, 1.5)	0.571	1.0 (−1.7, 3.9)	0.435	−1.0 (−4.0, 2.4)	0.609	0.4 (−0.2, 1.0)	0.196
LBNP ∗ NPPV	1.0 (0.1, 1.9)	0.033^#^	0.2 (−1.0, 1.4)	0.777	1.1 (−0.4, 2.3)	0.186	−0.1 (−0.4, 0.2)	0.504

	SV (mL)		HR (beats/min)		MAP (mm Hg)		PP (mm Hg)	
	Estimate (95% CI)	*P* value	Estimate (95% CI)	*P* value	Estimate (95% CI)	*P* value	Estimate (95% CI)	*P* value

LBNP	−11.2 (−11.8, −9.6)	<0.001^#^	7.3 (6.0, 8.6)	<0.001^#^	−0.4 (−1.5, 0.6)	0.415	−3.5 (−4.5, −2.0)	<0.001^#^
NPPV	−3.0 (−8.5, −1.3)	<0.009^#^	0.3 (−4.1, 4.7)	0.898	0.4 (−3.1, 4.0)	0.822	1.7 (−3.2, 5.7)	0.586
LBNP ∗ NPPV	−0.7 (−2.3, 0.7)	0.305	1.6 (−0.2, 3.5)	0.091	−0.7 (−2.2, 0.8)	0.347	−0.6 (−2.5, 1.3)	0.519

Estimates of LBNP-effects and LBNP ∗ NPPV-effects are given as changes per 20 mmHg change in LBNP (−20, −40, −60, and −80 mmHg). Estimates of NPPV-effects are constant and independent of LBNP-level. There are separate statistically significant effects of both LBNP and NPPV on SV. This means that LBNP led to a decrease in SV both during spontaneous breathing and NPPV, and NPPV led to a decrease in SV both during normo- and hypovolemia. In contrast, the effect of NPPV on ΔPP is due to a significant interaction between LBNP and NPPV (LBNP ∗ NPPV), which means that NPPV only affected ΔPP significantly during hypovolemia. PI is significantly affected by LBNP alone.

LBNP: lower body negative pressure; NPPV: noninvasive positive pressure ventilation; LBNP ∗ NPPV: interaction between LBNP and NPPV; ΔPP: pulse pressure variation; ΔPOP: photoplethysmographic waveform amplitude variation; PVI: pleth variability index; PI: perfusion index; SV: stroke volume; HR: heart rate; MAP: mean arterial pressure; PP: pulse pressure; ^#^: statistically significant.
